# Efficient derivation of extended pluripotent stem cells from NOD-*scid Il2rg*^−/−^ mice

**DOI:** 10.1007/s13238-018-0558-z

**Published:** 2018-06-13

**Authors:** Yaqin Du, Ting Wang, Jun Xu, Chaoran Zhao, Haibo Li, Yao Fu, Yaxing Xu, Liangfu Xie, Jingru Zhao, Weifeng Yang, Ming Yin, Jinhua Wen, Hongkui Deng

**Affiliations:** 1 Peking University Stem Cell Research Center, Department of Cell Biology, School of Basic Medical Sciences, Peking University Health Science Center, Beijing, 100191 China; 2 Peking University-Tsinghua University-National Institute of Biological Sciences Joint Graduate Program, College of Life Sciences, Peking University, Beijing, 100871 China; 3 The MOE Key Laboratory of Cell Proliferation and Differentiation, College of Life Sciences, Peking-Tsinghua Center for Life Sciences, Peking University, Beijing, 100871 China; 4 Beijing Vitalstar Biotechnology, Beijing, 100012 China

**Keywords:** extended pluripotent stem cell, NOD-*scid Il2rg*^−/−^ mice, embryonic and extraembryonic lineages, chemical reprogramming

## Abstract

Recently we have established a new culture condition enabling the derivation of extended pluripotent stem (EPS) cells, which, compared to conventional pluripotent stem cells, possess superior developmental potential and germline competence. However, it remains unclear whether this condition permits derivation of EPS cells from mouse strains that are refractory or non-permissive to pluripotent cell establishment. Here, we show that EPS cells can be robustly generated from non-permissive NOD-*scid Il2rg*^−/−^ mice through *de novo* derivation from blastocysts. Furthermore, these cells can also be efficiently generated by chemical reprogramming from embryonic NOD-*scid Il2rg*^−/−^ fibroblasts. NOD-*scid Il2rg*^−/−^ EPS cells can be expanded for more than 20 passages with genomic stability and can be genetically modified through gene targeting. Notably, these cells contribute to both embryonic and extraembryonic lineages *in vivo*. More importantly, they can produce chimeras and integrate into the E13.5 genital ridge. Our study demonstrates the feasibility of generating EPS cells from refractory mouse strains, which could potentially be a general strategy for deriving mouse pluripotent cells. The generation of NOD-*scid Il2rg*^−/−^ EPS cell lines permits sophisticated genetic modification in NOD-*scid Il2rg*^−/−^ mice, which may greatly advance the optimization of humanized mouse models for biomedical applications.

**Electronic supplementary material:**

The online version of this article (10.1007/s13238-018-0558-z) contains supplementary material, which is available to authorized users.

## Introduction

One major milestone in stem cell biology is the generation of mouse pluripotent stem cells (PSCs) from mouse blastocysts (Evans and Kaufman, 1981; Martin, 1981). These cells can self-renew *in vitro* and have the potential to differentiate into all the cell types constituting the body, thus providing invaluable tools for understanding mammalian development and generating genetically modified mouse models. To achieve wide applications of mouse PSCs in generating mouse models, one major goal in stem cell biology is to establish mouse PSCs from different genetic backgrounds. However, earlier studies revealed that the generation of mouse PSCs is highly strain-dependent (McWhir et al., 1996; Brook and Gardner, 1997), and only a few mouse strains such as 129 are permissive for PSC derivation using traditional conditions for culturing mouse PSCs (Kawase et al., 1994; McWhir et al., 1996; Brook and Gardner, 1997; Anderson et al., 2009).

Notably, recent significant advances in mechanistic understanding of pluripotency have led to the optimization of culturing medium for mouse PSCs (Buehr and Smith, 2003; Ying et al., 2003; Lodge et al., 2005; Bryja et al., 2006a; Bryja et al., 2006b; Umehara et al., 2007; Yang et al., 2009). One representative study is the development of a 2i/LIF medium, which supports the maintenance of mouse PSCs in the naïve pluripotent state (Ying et al., 2008). Importantly, the use of 2i/LIF medium has enabled successful derivation of mouse PSCs from several non-permissive mouse strains, such as mice with nonobese diabetic (NOD) background (Hanna et al., 2009; Nichols et al., 2009; Liu et al., 2015). However, recent studies have shown that prolonged culture of mouse pluripotent cells in 2i/LIF condition leads to significant impairment of epigenetic and genomic stability as well as of the developmental potential of these cells (Choi et al., 2017; Yagi et al., 2017). As a result, there is still a strong demand for establishing new culturing conditions that can capture mouse PSCs from a wide range of mouse strains.

Recently, our group reported a novel culture condition (human LIF, CHIR99021, (S)-(+)-dimethindene maleate and minocycline hydrochloride; LCDM) that supports the derivation and long-term culture of extended pluripotent stem (EPS) cells (Yang et al., 2017). EPS cells are characterized by expanded developmental potential to both embryonic (Em) and extraembryonic (ExEm) lineages. Furthermore, after long-term culturing, these cells possess normal karyotype and a robust ability to produce chimera and germline transmission as evidenced by single cell injection assay (Yang et al., 2017). Considering the superior developmental potency and stability of EPS cells, it is promising to investigate whether the LCDM condition supports generation of EPS cells from non-permissive mouse strains, which has not been explored yet.

To promote the wide applications of mouse EPS cells, another important question is whether these cells can be generated from somatic cells through reprogramming, thereby bypassing the use of mouse embryos. Remarkably, recently we have established a complete chemical approach to generate chemically-induced pluripotent stem cells (CiPSCs) from somatic cells (Hou et al., 2013; Zhao et al., 2015; Ye et al., 2016). In principle, compared to conventional transgenic methods (Takahashi and Yamanaka, 2006; Brambrink et al., 2008; Okita et al., 2008; Stadtfeld et al., 2008; Woltjen et al., 2009), this chemical approach is more favorable for generating EPS cells from somatic cells, because it circumvents the use of exogenous genetic factors. In this regard, it is important to explore the possibility of generating EPS cells from somatic cells through a complete chemical approach, which could become a more convenient way to establish EPS cells compared to *de novo* derivation from mouse embryos.

In this study, we sought to establish EPS cells from non-permissive NOD-*scid Il2rg*^−/−^ mice. These mice are highly immunodeficient and permit robust human cell engraftment (Ito et al., 2002; Shultz et al., 2005), conferring significant potential in generating humanized mouse models (Shultz et al., 2007; Ito et al., 2012). Using LCDM medium, we designed two approaches to generate NOD-*scid Il2rg*^−/−^ EPS cells: *de novo* derivation from blastocysts and chemical reprogramming from embryonic fibroblasts. We showed that EPS cells with normal karyotype could be robustly derived, which possess extended developmental potential to Em and ExEm lineages and robust chimeric ability. Our established NOD-*scid Il2rg*^−/−^ EPS cells would be a powerful tool for generating next-generation humanized mice.

## Results

### Generation of NOD-*scid Il2rg*^−/−^ extended pluripotent stem cells

**Figure 1:**
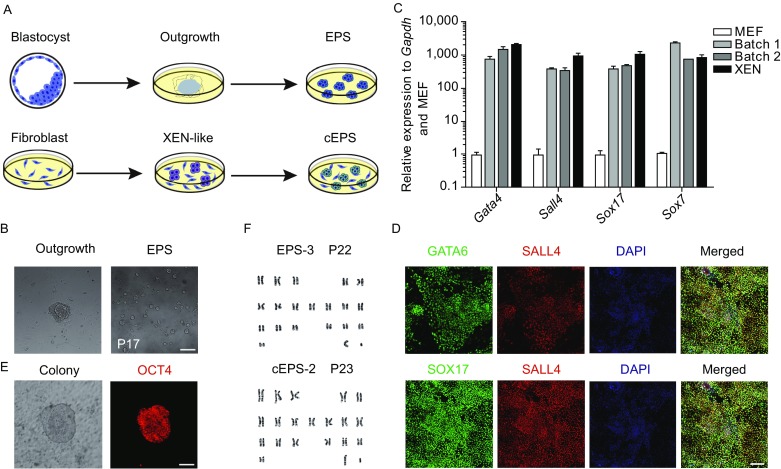
**Generation of NOD-*scid Il2rg***^**−/−**^ **extended pluripotent stem cells**. (A) Schematic of two approaches used for generating NOD-*scid Il2rg*^−/−^ extended pluripotent stem cells: *de novo* derivation from blastocysts (upper panels) and chemical reprogramming from embryonic fibroblasts (lower panels). (B) Phase-contrast images of *de novo* derived outgrowth and EPS colonies for 17 passages in LCDM medium. Scale bars, 100 μm. (C) qRT-PCR analysis of XEN marker genes expression during the chemical induction process (day 16). Error bars indicate SEM (*n* = 2). (D) Co-immunostaining of XEN marker genes during the chemical induction process (day 16). Upper panels: GATA6 and SALL4; lower panels: SOX17 and SALL4. Scale bars, 100 μm. (E) Immunofluorescence of OCT4-positive primary colonies at the end of the chemical induction (day 40). Scale bars, 100 μm. (F) Typical karyotypes of EPS (passage 22) and cEPS (passage 23) cells. Each cell line counts 30 cells

To derive NOD-*scid Il2rg*^−/−^ extended pluripotent stem cells, we tried to establish EPS cells by *de novo* derivation from mouse blastocysts and chemical induction from embryonic fibroblasts (Hou et al., 2013; Zhao et al., 2015; Ye et al., 2016) (Fig. 1A). Initially, a total of 30 embryonic day 3.5 (E3.5) blastocysts were isolated from NOD-*scid Il2rg*^−/−^ pregnant female mice, which were further seeded individually onto feeder cells using the LCDM medium (Yang et al., 2017). After 5–8 days, 28 outgrowths were formed, which were single-cell passaged onto new feeder cells. After further passaging, 20 cell lines were established with undifferentiated, rounded morphology and normal karyotype, which were further passaged more than 20 times (Fig. 1B and 1F). These results suggest successful derivation of EPS cells from NOD-*scid Il2rg*^−/−^ blastocysts.

Next, we attempted to induce E13.5 NOD-*scid Il2rg*^−/−^ mouse fibroblast into PSCs using small-molecule compounds, which were reported to successfully induce PSCs from mouse somatic cells in our previous studies (Hou et al., 2013; Zhao et al., 2015; Ye et al., 2016). Consistent with our previous reports, during the chemical induction process, extra-embryonic endoderm (XEN)-like cells could be induced before the CiPSC primary colony formation. By using qRT-PCR and immunofluorescence analysis, at the end of stage 1 (day 16), we found that the XEN-like cells expressed representative XEN markers, such as *Gata4, Sall4, Sox7* and *Sox17*, at the mRNA and protein levels (Fig. 1C and 1D). We further induced the XEN-like cells into PSCs by changing the reprogramming medium from stage 2 to stage 3. At day 40, domed colonies were formed with apparent OCT4 expression, which were further isolated and cultured in LCDM medium (Fig. 1E). A total of 9 chemically-induced EPS (cEPS) cell lines were established using this chemical reprogramming method, which were cultured long-term for more than 20 passages while maintaining a normal karyotype (Fig. 1F). Notably, the genetic identities of the established NOD-*scid Il2rg*^−/−^ EPS and cEPS cell lines were confirmed by detecting specific mutant gene sites in protein kinase, DNA activated, catalytic polypeptide *(Prkdc)* and the interleukin-2 receptor γ-chain gene (*Il2rg*) locus using genomic PCR (Fig. S1A). Collectively, these data indicate that extended pluripotent stem cells with NOD-*scid Il2rg*^−/−^ genetic background could be established by *de novo* derivation from blastocysts and chemical reprogramming from fibroblasts.

### NOD-*scid Il2rg*^−/−^ EPS and cEPS cells possess pluripotent features

**Figure 2:**
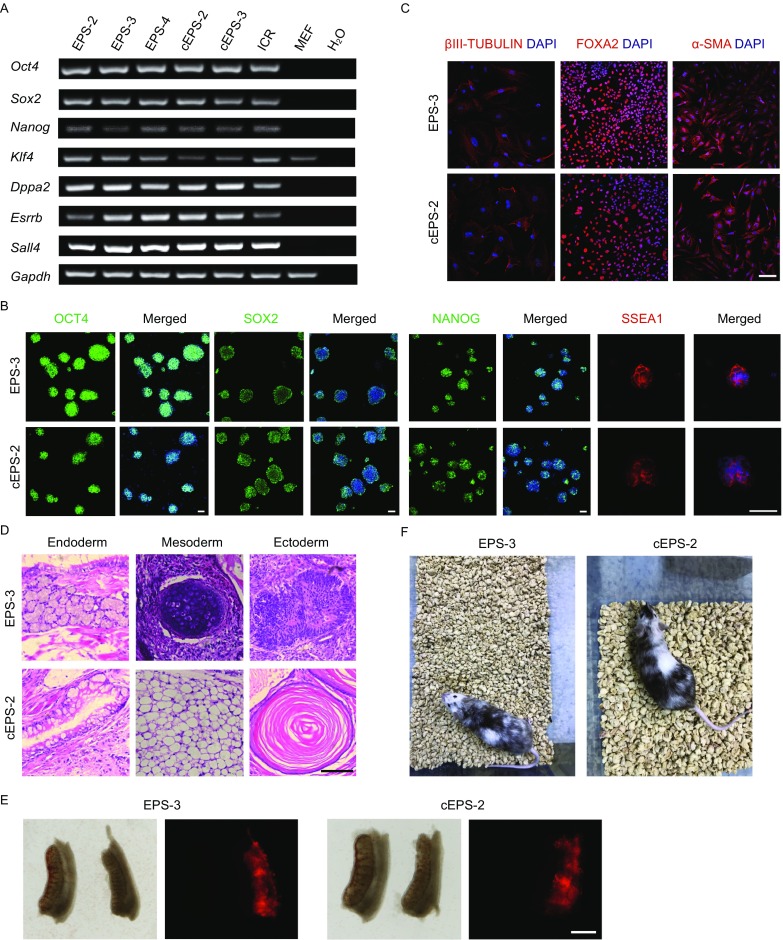
**Characterization of pluripotent features of NOD-*scid Il2rg***^**−/−**^ **EPS and cEPS cells**. (A) RT-PCR analysis of pluripotent gene expression in NOD-*scid Il2rg*^−/−^ EPS and cEPS cells. EPS-2, EPS-3, EPS-4: NOD-*scid Il2rg*^−/−^ EPS cell lines; cEPS-2, cEPS-3: NOD-*scid Il2rg*^−/−^ cEPS cell lines; ICR: ICR strain EPS cell line; MEF: NOD-*scid Il2rg*^−/−^ mouse embryonic fibroblasts. (B) Immunofluorescence of representative pluripotent markers in NOD-*scid Il2rg*^−/−^ EPS and cEPS cells. Scale bar, 50 μm. (C) Immunofluorescence of representative markers of the three germ lineages in NOD-*scid Il2rg*^−/−^ EPS and cEPS cells during *in vitro* EB differentiation. Scale bar, 100 μm. (D) Hematoxylin and eosin staining of NOD-*scid Il2rg*^−/−^ EPS and cEPS derived teratomas. Scale bar, 100 μm. (E) Representative images showing the extensive integration of TD^+^ EPS/cEPS cells into the E13.5 genital ridge. Scale bar, 400 μm. (F) Postnatal chimeras generated by injection of NOD-*scid Il2rg*^−/−^ EPS and cEPS cells into C57BL/6 8-cell embryo

We next examined the pluripotent features of NOD-*scid Il2rg*^−/−^ EPS and cEPS cells using RT-PCR and immunofluorescence. Typical pluripotent marker genes, such as *Oct4*, *Sox2*, *Nanog*, *Klf4*, *Dppa2*, *Esrrb* and *Sall4*, were expressed in EPS and cEPS cells at the mRNA level, but not in the control NOD-*scid Il2rg*^−/−^ fibroblasts (Figs. 2A and S1C). In addition, the protein expression of OCT4, NANOG, SOX2 and SSEA-1 in NOD-*scid Il2rg*^−/−^ EPS and cEPS cells were also confirmed (Fig. 2B). We next examined their differentiation potential using embryonic body (EB) differentiation analysis. Immunostaining of EB-derived cells showed that these cells expressed α-SMA (mesoderm marker), βIII TUBULIN (ectoderm marker) and FOXA2 (endoderm marker) (Fig. 2C). To further examine the pluripotency of NOD-*scid Il2rg*^−/−^ EPS and cEPS cells *in vivo*, we employed teratoma formation analysis. Histological analysis of teratomas derived from NOD-*scid Il2rg*^−/−^ EPS and cEPS cells showed the presence of lineages from different germ layers, including cartilage, adipose, muscle, neural tissue, columnar epithelium and epidermis, indicating that these cells could further differentiate *in vivo* (Fig. 2D). Taken together, these data suggest that NOD-*scid Il2rg*^−/−^ EPS and cEPS cells possess features of pluripotency.

We next performed chimera assays using NOD-*scid Il2rg*^−/−^ EPS and cEPS cells. To label these cells, a construct containing Ef1α-*Tdtomato* was knocked into the *Rosa26* locus, which permits expression of TDTOMATO (Fig. S1D and S1E). To investigate whether NOD-*scid Il2rg*^−/−^ EPS and cEPS cells are germline-competent, TDTOMATO-positive (TD^+^) colonies were picked up and injected into eight-cell embryos. 40 eight-cell embryos were injected with EPS cells while 36 eight-cell embryos were injected with cEPS cells. The injected embryos were then transplanted into pseudopregnant recipients. A portion of recipients were analyzed at E13.5, when the genital ridge was isolated for analysis. We found that TD^+^ cells robustly contributed to the germline (Fig. 2E). Furthermore, we also obtained postnatal chimeric mice derived from NOD-*scid Il2rg*^−/−^ EPS and cEPS cells (Fig. 2F). Taken together, these results suggest that NOD-*scid Il2rg*^−/−^ EPS and cEPS cells are not only capable of producing chimera, but also of contributing to the germline, confirming their pluripotency *in vivo*.

### NOD-*scid Il2rg*^−/−^ EPS and cEPS cells can contribute to both embryonic and extraembryonic lineages

**Figure 3:**
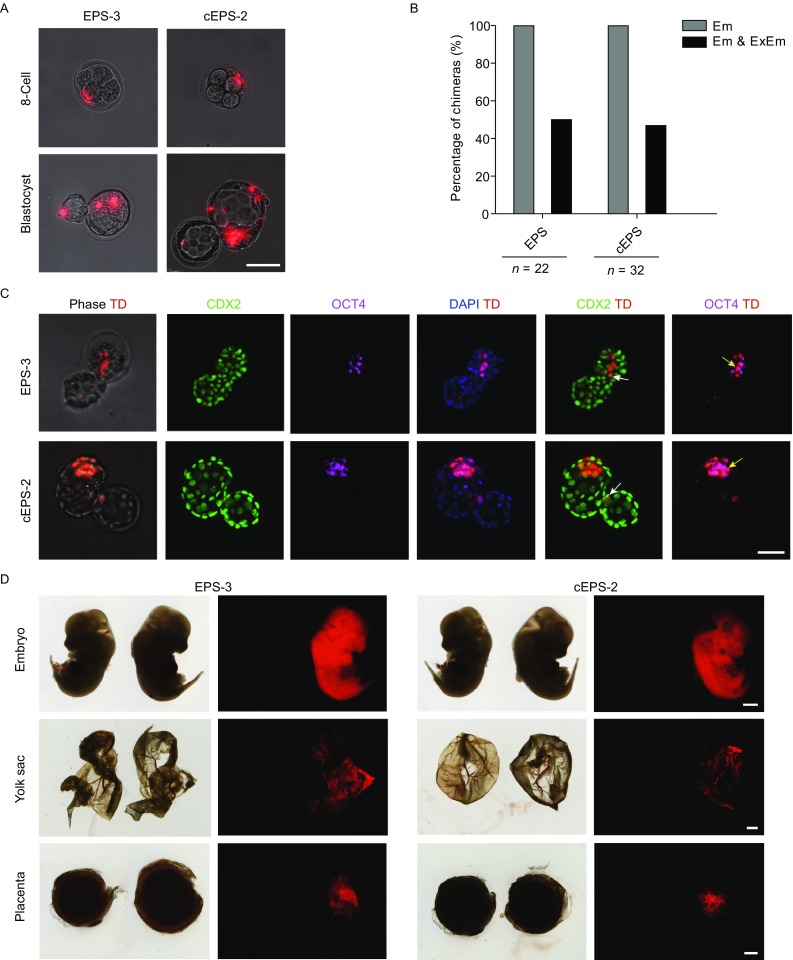
**NOD-*scid Il2rg***^**−/−**^ **EPS and cEPS cells can contribute to both embryonic and extraembryonic lineages**. (A) Representative images of TD^+^ EPS or cEPS cells injected embryo, with the localization of TD^+^ EPS/cEPS derivatives in both Em and ExEm segments after injection. Scale bar, 50 μm. (B) Bar chart showing the percentage of chimeras after blastocyst hatch. Em & ExEm, NOD-*scid Il2rg*^−/−^ EPS or cEPS cells integrating into both Em & ExEm lineages of mouse embryo. Em, NOD-*scid Il2rg*^−/−^ EPS or cEPS integrating into only Em of mouse embryo *in vitro*. *n* indicates numbers of hatched blastocysts. (C) Representative images showing the immunostaining of NOD-*scid Il2rg*^−/−^ EPS or cEPS-derived chimeric blastocysts (hatching) with antibodies specific to ICM (OCT4) and TE (CDX2). DAPI stains the nucleus. The arrows indicate CDX2^+^TD^+^ and OCT4^+^TD^+^ donor cells. Scale bar, 50 μm. (D) Representative images of E13.5 embryo after injection of TD^+^ EPS or cEPS cells, which contribute to embryo, placenta and yolk sac tissues. Negative control: wild-type fetus. Scale bar, 1 mm

To examine the expanded developmental potency of NOD-*scid Il2rg*^−/−^ EPS and cEPS cells, we injected TD^+^ NOD-*scid Il2rg*^−/−^ EPS and cEPS cells into eight-cell mouse embryos (Fig. 3A). The injected mouse embryos were then cultured in KSOM medium for 48–72 h *in vitro*. Surviving TD^+^ cells were integrated into inner cell mass (ICM) of all hatched embryos. Notably, we observed that TD^+^ cells contributed to both ICM and trophectoderm (TE) lineages in 50% (11/22) of embryos with NOD-*scid Il2rg*^−/−^ EPS cell injection and in 46.9% (15/32) of embryos with NOD-*scid Il2rg*^−/−^ cEPS cell injection (Fig. 3A and 3B). Immunofluorescence analysis further confirmed the expanded potential of NOD-*scid Il2rg*^−/−^ EPS and cEPS cells to both Em and ExEm lineages, as the chimeric TD^+^ cells expressed either CDX2 or OCT4 in TE or ICM segments respectively (Fig. 3C). These data demonstrate that NOD-*scid Il2rg*^−/−^ EPS and cEPS cells have the capacity to develop toward both ICM and TE lineages in preimplantation embryos.

To further analyze the bi-directional developmental potentials, we transferred the injected eight-cell embryos into the oviduct of recipient mice. E13.5 embryos were recovered to examine the chimeric contributions of TD^+^ cells in Em and ExEm segments. Consistent with chimerism of TD^+^ cells in the TE and ICM segments in the blastocysts, TD^+^ EPS derivatives were integrated not only in the Em lineages but also ExEm tissues, including yolk sac and placenta (Figs. 3D and S1F). Collectively, these data suggest that the NOD-*scid Il2rg*^−/−^ EPS and cEPS cells possess the capacity to contribute to both Em and ExEm lineages.

### Further analysis of extended developmental potency of LCDM cultured NOD-*scid Il2rg*^−/−^ EPS and cEPS cells

**Figure 4:**
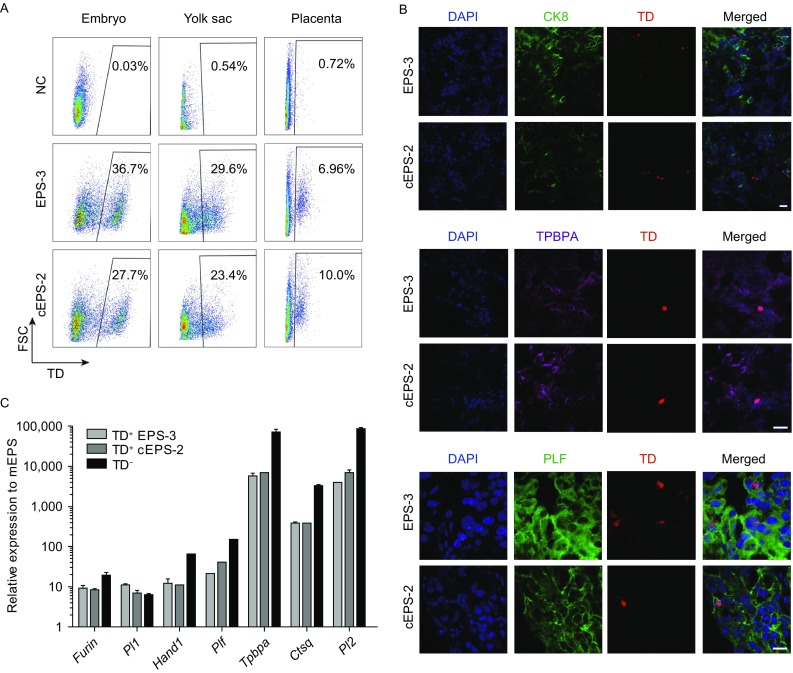
**Further characterization of chimeric capacity of NOD-*scid Il2rg***^**−/−**^ **EPS and cEPS cells**. (A) FACS detection of the percentage of NOD-*scid Il2rg*^−/−^ EPS or cEPS-derived TD^+^ cells in E13.5 fetus, including embryo, yolk sac and placenta. Negative control: wild-type fetus. (B) Representative images showing NOD-*scid Il2rg*^−/−^ EPS or cEPS-derived cells contributing to trophoblastic lineages in chimeric E17.5 placentas. The TD^+^ cells were co-stained with trophoblast marker CK8, trophoblast giant cells marker PLF and spongiotrophoblas marker TPBPA. Scale bars, 20 μm. (C) qRT-PCR analysis of placenta specific genes in TD^+^ and TD^−^ placenta cells in E17.5 placentas. Expression levels normalized to EPS cells. Error bars indicate SEM (*n* = 2)

To further explore the chimeric capacity of NOD-*scid Il2rg*^−/−^ EPS and cEPS cells, we collected three types of tissues (embryo, yolk sac and placenta) from recipient mice for analysis. Fluorescence activated cell sorting (FACS) analysis was first performed to quantitatively verify the contribution of the TD^+^ NOD-*scid Il2rg*^−/−^ EPS and cEPS derivatives in E13.5 embryos, yolk sac and placenta (Fig. 4A). Robust presence of TD^+^ NOD-*scid Il2rg*^−/−^ EPS derivatives was observed in the embryo (36.7%), yolk sac (29.6%) and placenta (6.96%). Similar results were also observed with the NOD-*scid Il2rg*^−/−^ cEPS chimeras (27.7% of chimerism in the embryo, 23.4% in the yolk sac and 10.0% in the placenta). We next analyzed whether the chimeric TD^+^ cells adopt an ExEm fate in the placenta using immunofluorescence. Notably, we detected the expression of representative trophoblast marker cytokeratin 8 (CK8) in these chimeric cells (Fig. 4B). Moreover, we also observed the expression of TPBPA and PLF, typical markers for spongiotrophoblast and trophoblast giant cells, in these chimeric cells (Fig. 4B). To further confirm the expression of trophoblast markers in EPS and cEPS derivatives in the placenta, we sorted the TD^+^ cells from E17.5 placental tissues for qRT-PCR analysis. Importantly, multiple trophoblast markers, such as *Furin*, *Pl1*, *Hand1*, *Plf*, *Tpbpa* and *Pl2*, were significantly upregulated compared to the original EPS and cEPS cells (Fig. 4C). These data further verified the extended developmental potency of NOD-*scid Il2rg*^−/−^ EPS and cEPS cells.

### Genetic manipulation of NOD-*scid Il2rg*^−/−^ EPS cells in LCDM condition

To assess the feasibility of performing genetic manipulation in NOD-*scid Il2rg*^−/−^ EPS cells in LCDM condition, we inserted the human interleukin 6 (*IL-6*) gene sequence into the mouse Il6 locus in NOD-*scid Il2rg*^−/−^ EPS cells (Fig. S2A), which could improve the human T- and B-cell engraftment and differentiation (Yu et al., 2017). Targeted NOD-*scid Il2rg*^−/−^ EPS colonies were identified by genomic PCR (Fig. S2B) and then expanded to establish stable human *IL-6* inserted NOD-*scid Il2rg*^−/−^ EPS cell lines (Fig. S2C). To further explore whether human *IL-6* is faithfully expressed *in vivo*, we injected the human *IL-6* inserted NOD-*scid Il2rg*^−/−^ EPS cells into C57BL/6 mouse embryos and obtained chimeric mice successfully (Fig. S2D). ELISA measurement of human IL-6 protein level in the chimeric mice was detected after lipopolysaccharide (LPS) stimulation, which could increase IL-6 level in circulatory system. We observed that the human IL-6 protein level increased from 0.520 pg/mL to 48.700 pg/mL, which was significantly higher than that in the control group (Fig. S2E). Collectively, these data indicate that NOD-*scid Il2rg*^−/−^ EPS cells enable efficient genetic manipulation.

## Discussion

In this study, we show that EPS cells can be established efficiently from the NOD-*scid Il2rg*^−/−^ mouse strain using the LCDM medium. NOD-*scid Il2rg*^−/−^ EPS cells possess pluripotent features, Em and ExEm developmental potentials, a robust chimeric formation ability and germline competence. Furthermore, these cells can be stably expanded during long-term culturing and also genetically modified in a robust manner through gene targeting. These features of NOD-*scid Il2rg*^−/−^ EPS cells make them a promising resource for generating optimized humanized mouse models through sophisticated genetic modification.

Importantly, the generation of NOD-*scid Il2rg*^−/−^ EPS cells provides proof-of-principle that the LCDM condition permits robust derivation of EPS cells from non-permissive or refractory mouse strains. The LCDM condition has been reported to support derivation of EPS cell lines with C57BL/6×C57BL/6 or C57BL/6×129 backgrounds (Yang et al., 2017). As the efficiency of deriving mouse embryonic stem (ES) cells is highly strain-dependent (McWhir et al., 1996; Brook and Gardner, 1997), it is important to investigate whether the LCDM condition enables EPS cell derivation from non-permissive strains such as mice with NOD background, which are refractory to ES cell derivation using traditional ES cell culturing medium (Nagafuchi et al., 1999; Brook et al., 2003). Notably, the efficiency of deriving EPS cells from NOD-*scid Il2rg*^−/−^ blastocysts was 66.7%, which is significantly higher than previous reports (Liu et al., 2015). This result in combination with our previous report supports the notion that the LCDM condition permits EPS cell derivation from different mouse strains, including permissive and non-permissive backgrounds (Yang et al., 2017).

Another significant finding is that EPS cells can be efficiently derived from NOD-*scid Il2rg*^−/−^ fibroblasts using a completely chemical based reprogramming approach. While we demonstrated the induction of CiPSCs from mouse somatic cells in our previous studies (Hou et al., 2013; Ye et al., 2016; Zhao et al., 2015), it is unclear whether this chemical approach can be applied to different mouse strains, especially non-permissive strains, or to the induction of EPS cells. Notably, we have obtained EPS cells from multiple mouse strains using complete chemical approach (Fig. S1G). Consistent with our previous reports (Hou et al., 2013; Zhao et al., 2015; Ye et al., 2016), the induction of cEPS cells from NOD-*scid Il2rg*^−/−^ fibroblasts occurs via the XEN-like state (Fig. 1C and 1D), followed by the emergence of OCT4-positive primary colonies in 2i/LIF medium during stage 3 (Fig. 1E). Notably, when we tried to induce cEPS cells directly using the LCDM condition at stage 3, ES-like primary colonies were formed but failed to be passaged (data not shown). Instead, we found that the treatment of 2i/LIF medium was required for cEPS cell induction at the beginning of stage 3, and cEPS cells can be expanded after transferring the primary ES-like colonies to the LCDM condition. Accordingly, to chemically induce EPS cells, the final stage of reprogramming requires the treatment of 2i/LIF condition to activate the pluripotent regulatory network (Hou et al., 2013; Zhao et al., 2015; Ye et al., 2016). On the other hand, because the LCDM condition was used as the final condition for culturing the reprogramed CiPSCs but not used as a reprogramming condition, the reprogramming efficiency is not influenced by the use of LCDM medium in our study. In brief, our results demonstrate the feasibility of generating EPS cells using the complete chemical approach from non-permissive strains.

Finally, our established NOD-*scid Il2rg*^−/−^ EPS cell lines can be stably expanded long-term using the LCDM condition. The analyzed EPS cells in the LCDM medium had normal karyotypes at approximately the 20th passage (Fig. 1F). In contrast, NOD-*scid Il2rg*^−/−^ ES cells showed abnormal chromosome structure under the 2i/LIF condition (Fig. S1B), which is consistent with recent reports (Choi et al., 2017; Yagi et al., 2017). The genetic stability and germline competence of NOD-*scid Il2rg*^−/−^ EPS cells (Figs. 1F and 2E), in combination with efficient gene targeting in these cells (Fig. S2), makes it possible to perform sophisticated genetic modifications in NOD-*scid Il2rg*^−/−^ mice for developing better humanized mouse models in the future.

In summary, our study demonstrates the feasibility of generating EPS cell lines from “non-permissive” mouse strains by directly derivation from mouse blastocysts or chemical reprogramming. The established NOD-*scid Il2rg*^−/−^ EPS cell lines provide a powerful tool for further optimizing current humanized mouse models, which holds great promise in biomedical applications. More importantly, our findings suggest that deriving EPS cells could be a general strategy to establish pluripotent cell lines from different mouse strains, which have great applicative potentials for studying mammalian development and generating mouse models.

## Materials and methods

### Mice

NOD-*scid Il2rg*^−/−^ mice were provided by VITALSTAR Biotechnology Co., Ltd. C57BL/6, ICR and nude mice were obtained from Peking University Health Science Center. C57BL/6J-Tg 11Imeg/Rbrc (OG) mice (Stock No.: RBRC00771) were obtained from RIKEN BioResource Center. All the mouse work was done with the approval of Institutional Animal Care and Use Committee of Peking University Health Science Center.

### Establish NOD-*scid Il2rg*^−/−^ EPS cells from blastocysts

E3.5 blastocysts were collected from uterus of pregnant NOD-*scid Il2rg*^**−/−**^ mice and seeded on mitomycin C (Sigma-Aldrich,M4287)-treated mouse embryonic fibroblast (MEF) feeder cells with LCDM medium containing DMEM/F12 (Thermo Fisher Scientific, 11330-032), Neurobasal (ThermoFisher Scientific, 21103-049), 0.5% N2 supplement (Thermo Fisher Scientific, 17502-048), 1% B27 supplement (Thermo Fisher Scientific, 12587-010), 1% GlutaMAX (Thermo Fisher Scientific, 35050-061), 1% nonessential amino acids (Thermo Fisher Scientific, 11140-050), 1% penicillin-streptomycin (Thermo Fisher Scientific, 15140-122), 0.1 mmol/L β-mercaptoethanol (Thermo FisherScientific, 21985-023), 10 ng/mL recombinant human LIF (Peprotech, 300-05), 3 μmol/L CHIR99021 (Tocris, 4423), 2 μmol/L (S)-(+)-dimethindenemaleate (Tocris, 1425), 2 μmol/L minocycline hydrochloride (Santa Cruz Biotechnology, sc-203339). After 5–8 days, outgrowths were picked up and digested by 0.05% trypsin-EDTA (Thermo Fisher Scientific, 25300-062). The digested single cells were plated on new MEF feeders with LCDM medium. EPS colonies appeared about 4 days later. EPS cells were single-cell passaged every 3 days using 0.05% trypsin-EDTA and used for further characterization.

### Chemical induction of cEPS cells from fibroblasts

#### Culture medium preparation

Stage 1: For day 0–12, KnockOut DMEM (Thermo Fisher Scientific, 10829-018) supplemented with 10% KSR (Thermo Fisher Scientific, 10828-028), 10% fetal bovine serum (FBS) (PAN-biotech,P30-3302), 1% GlutaMAX, 1% nonessential amino acids, 0.1 mmol/L β-mercaptoethanol, 1% penicillin-streptomycin, 100 ng/mL bFGF (Origene, TP750002) containing the small-molecule cocktail: 0.5 mmol/L VPA, 20 μmol/L CHIR99021, 10 μmol/L 616452, 5 μmol/L tranylcypromine, 10 μmol/L forskolin, 0.05 μmol/L AM580 and 5 μmol/L EPZ004777. For day 12–16, the concentration of bFGF and CHIR99021 were reduced to 25 ng/mL and 10 μmol/L respectively.

Stage 2: For day 16–28, KnockOut DMEM supplemented with 10% KSR, 10% FBS, 1% GlutaMAX, 1% nonessential amino acids, 0.1 mmol/L β-mercaptoethanol, 1% penicillin-streptomycin, 25 ng/mL bFGF containing the small-molecule cocktail: 0.5 mmol/L VPA, 10 μmol/L CHIR99021, 10 μmol/L 616452, 5 μmol/L tranylcypromine, 10 μmol/L forskolin, 0.05 μmol/L AM580, 0.05 μmol/L DZNep, 0.5 μmol/L 5-aza-dC and 5 μmol/L SGC0946.

Stage 3: Neurobasal and DMEM/F12 mixed equally supplemented with 1% GlutaMAX, 1% nonessential amino acids, 0.1 mmol/L β-mercaptoethanol, 1% penicillin- streptomycin, 1% N2 supplement, 2% B27 supplement, 1000 U/mL LIF, 3 μmol/L CHIR99021, 1 μmol/L PD0325901.

#### Chemical induction of CiPSCs

The primary MEFs were seeded at a density of 100,000 cells per well of a 6-well plate. After 12 h, the original medium was replaced with stage 1 medium (day 0), which is changed every 4 days. On day 12, the cells were trypsinized by 0.25% trypsin-EDTA (Thermo Fisher Scientific, 25200-114) and replated at a density of 100,000–200,000 cells per well of a 6-well plate with the stage 1 medium (having reduced the concentration of bFGF and CHIR99021). On day 16, the culture condition was transformed into stage 2 medium and the medium was changed every 2–4 days. On day 28, stage 3 medium was added into the plate and changed every 2–4 days. Around day 40, the ES cell-like colonies were large enough and could be picked up for expansion.

The primary colonies were picked up and digested by 0.25% trypsin-EDTA for 5 min. Then, the digested CiPSCs were re-suspended with LCDM medium and re-plated on new MEF feeders. Medium was changed every day. The cells were passaged every 3–6 days using 0.05% trypsin-EDTA at a ratio of 1:3 to 1:10 (depending on the rate of propagation). After a few passages in LCDM medium, cells could gradually proliferate well and obtain the extended pluripotent characteristics. cEPS cells were used for long-term culture and further characterization *in vivo* and *in vitro*. Detail information of small molecules used in chemical reprogramming is listed in Table S3.

### Immunofluorescence

Samples were fixed in 4% paraformaldehyde for 15 min at room temperature, followed with being blocked and permeabilized in PBS (Corning, 21-040-CVR) with 0.25% Triton X-100 (Sigma-Aldrich, T8787) and 3% donkey serum (Jackson Immuno Research, 017-000-121) for 30 min at room temperature. Then, samples were incubated with primary antibody at 4 °C overnight, followed with being incubated with secondary antibody for 1 h at room temperature. DAPI (Roche Life Science, 10236276001) was used to stain the nucleus. Antibodies used are listed in Table S2.

### EB formation

NOD-*scid Il2rg*^−/−^ EPS cells cultured on matrigel (Corning, 354248)-coated plates were digested into single cells by 0.05% trypsin-EDTA and seeded on ultra-low attachment 24-well plate with about 5,000 cells per well in medium IMDM (Thermo Fisher Scientific, 12440-053) containing 15% FBS and 1% penicillin-streptomycin. Then 6 days later, the EB spheres were collected and plated on matrigel-coated 24-well plates in culture medium above for further growth. After 6 days, the cells were fixed for immunofluorescence.

### Teratoma formation

NOD-*scid Il2rg*^−/−^ EPS cells cultured on matrigel-coated plates were digested into single cells by 0.05% trypsin-EDTA and re-suspended in PBS with 0.1% matrigel. About 10 million cells were injected subcutaneously into 6-weeks nude mice, which were killed when the diameter of tumor reached 1.5 cm after 8-weeks growth. The teratomas were then embedded in paraffin and processed for hematoxylin and eosin staining.

### RT-PCR, qRT-PCR and genomic PCR

Total RNA was isolated from cells using Direct-zol RNA Miniprep (ZYMO RESEARCH, R2052). cDNA was prepared using TransScript First-Strand cDNA Synthesis SuperMix (TransGen Biotech, L20904), as the template of RT-PCR and qPCR. RT-PCR analysis performed using 2× Taq PCR StarMix (GenStar, A012-101), followed with gel electrophoresis. qPCR analysis was performed using KAPA SYBR FAST qPCR Master Mix (KAPA Biosystems, KM4100) on Bio-Rad CFX384TM Real-Time System. Gene expression normalized to *Gapdh* was analyzed using ΔΔCt method. For genomic PCR, genomic DNA was extracted with the DNeasy Blood & Tissue Kit (QIAGEN, 69506), DNA was amplificated by 2× Taq PCR StarMix. The primers used are listed in Table S1.

### Karyotype analysis

The analyzed cell lines were seeded on the matrigel-coated 25 cm^2^ tissue culture flask (Falcon, 6262022) at a ratio of 1:5 to 1:8, and sampled until the colonies reached 70% ± 80% confluence. The following chromosomal analysis was performed by the Peking University Center of Medical Genetics.

### Plasmid construction and cell transfection

NOD-*scid Il2rg*^−/−^ EPS cells were labeled by TDTOMATO fluorescence protein using CRISPR/Cas9 method. gRNAs was designed using CRISPR tool (http://crispr.mit.edu) and cloned into pX330 vector (Addgene, 42230). The sequence of optimized gRNA is 5′-CTAATGAGCCACTATGGATG-3′. The expression cassette containing Ef1α promoter-Tdtomato-SV40 polyA-LoxP-PGK promoter-Puro-bGH polyA-LoxP was constructed into pEASY-Blunt vector (TransGen Biotech, CB501) for targeting Rosa26 locus. Before transfection, the targeting vector was linearized by PvuI (New England BioLabs, R3150L). The primers used are listed in Table S1.

A total of 2 × 10^6^ cells were mixed with 10 μg of modified pX330 plasmid and 10 μg of targeting vector in final volume of 100 μL containing 82 μL P3 primary cell solution and 18 μL supplement 1 (PBP3-02250). The process of nucleofection used 4D-Nucleofector device (Lonza) according to the manufacturer’s instructions. The nucleofected cells were cultured on matrigel-coated plates and treated with 800 ng/μL puromycin dihydrochloride (Thermo Fisher Scientific, A1113803) after 60 h. Two days later, the TD^+^ colonies were picked up and passaged by 0.05% trypsin-EDTA. The cells were cultured at 37 °C in a 5% CO_2_ incubator and passaged every 3 days.

Human *IL-6* sequence was inserted in NOD-*scid Il2rg*^–/–^ EPS cells using the CRISPR/Cas9 method. The sequence of optimized gRNA is 5′-GTCTCAATAGCTCCGCCAGA-3′, the expression cassette containing human IL-6- SV40 polyA-LoxP-PGK promoter-Puro-bGH polyA-LoxP was constructed into pEASY-Blunt vector for targeting mouse *Il-6* locus. Correct inserted colonies were identified by genomic PCR and sequencing. The primers used are listed in Table S1.

### Chimeric assay

For NOD-*scid Il2rg*^−/−^ EPS and cEPS injection, cells were digested into single cell with 0.05% trypsin-EDTA, filtered through a 40 μm cell strainer and placed on the ice before injection. 4–10 cells were injected into 8-cell stage embryo of C57BL/6×C57BL/6 and 16–18 injected embryos were transferred into the oviduct of day 0.5 pseudopregnant ICR mouse. Fetuses were dissected at E13.5/E17.5 or grew until birth to identify the chimerism.

### Flow cytometry

Embryo, yolk sac and placenta samples from EPS/cEPS chimeras and wild type fetus at E13.5/E17.5 were cut into fragments and digested with collagenase IV (Thermo Fisher Scientific, 17104019) for 15 min. Then, most of tissues were dissociated into single cells by carefully pipetting up and down and filtered through a 40 μm cell strainer. All samples were analyzed on a BD LSRFortessa cell analyzer.

### Detection of placenta marker genes

After digestion of E17.5 EPS/cEPS-chimeric placenta, TD^+^ and TD^−^ placental cells were sorted by FACS and then extracted the total RNA using Trizol (Sigma), respectively. Smart-seq2 methods (Picelli et al., 2014) were performed for the limited RNA reverse transcription and amplification followed with qRT-PCR analysis. The dilution multiple of the samples was determined according to the expression of the internal reference and Ct values were stable at 18 to 19. The primers used are listed in Table S1.

### ELISA

Human *IL-6* inserted NOD-*scid Il2rg*^−/−^ EPS cells derived chimera mouse was used to test the human IL-6 expression. Plasma concentration of human IL-6 was measured using a human IL-6 ELISA Kit (Dakewe, DKW12-1060-096) according to the manufacturer’s instructions. 30 μg lipopolysaccharide (LPS, Invitrogen) was injected intraperitoneally into chimera and the plasma was harvested 2 h later.

## Electronic supplementary material

Below is the link to the electronic supplementary material.

Supplementary material 1 (PDF 60 kb)
