# Correction to: Efficient derivation of extended pluripotent stem cells from NOD-***scid Il2rg***^**−/−**^ mice

**DOI:** 10.1007/s13238-018-0571-2

**Published:** 2018-08-10

**Authors:** Yaqin Du, Ting Wang, Jun Xu, Chaoran Zhao, Haibo Li, Yao Fu, Yaxing Xu, Liangfu Xie, Jingru Zhao, Weifeng Yang, Ming Yin, Jinhua Wen, Hongkui Deng

**Affiliations:** 1 Peking University Stem Cell Research Center, Department of Cell Biology, School of Basic Medical Sciences, Peking University Health Science Center, Beijing, 100191 China; 2 Peking University-Tsinghua University-National Institute of Biological Sciences Joint Graduate Program, College of Life Sciences, Peking University, Beijing, 100871 China; 3 The MOE Key Laboratory of Cell Proliferation and Differentiation, College of Life Sciences, Peking-Tsinghua Center for Life Sciences, Peking University, Beijing, 100871 China; 4 Beijing Vitalstar Biotechnology, Beijing, 100012 China

## Correction to: Protein Cell 10.1007/s13238-018-0558-z

In the original publication Fig. 1D and supplementary material is incorrect. The correct Fig. 1D and supplementary material is provided in this correction.

**Figure 1:**
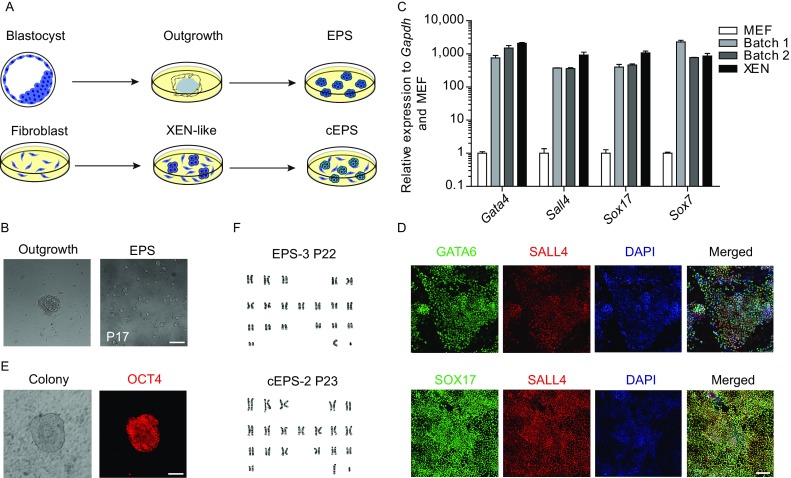
**Generation of NOD-*scid Il2rg***^**−/−**^ **extended pluripotent stem cells**. (A) Schematic of two approaches used for generating NOD-*scid Il2rg*^−/−^ extended pluripotent stem cells: *de novo* derivation from blastocysts (upper panels) and chemical reprogramming from embryonic fibroblasts (lower panels). (B) Phase-contrast images of *de novo* derived outgrowth and EPS colonies for 17 passages in LCDM medium. Scale bars, 100 μm. (C) qRT-PCR analysis of XEN marker genes expression during the chemical induction process (day 16). Error bars indicate SEM (*n* = 2). (D) Co-immunostaining of XEN marker genes during the chemical induction process (day 16). Upper panels: GATA6 and SALL4; lower panels: SOX17 and SALL4. Scale bars, 100 μm. (E) Immunofluorescence of OCT4-positive primary colonies at the end of the chemical induction (day 40). Scale bars, 100 μm. (F) Typical karyotypes of EPS (passage 22) and cEPS (passage 23) cells. Each cell line counts 30 cells

## Electronic supplementary material

Below is the link to the electronic supplementary material.

Electronic supplementary material 1 (PDF 170 kb)

Electronic supplementary material 2 (PPTX 62 kb)

