# Supplementary material for: Efficient derivation of extended pluripotent stem cells from NOD-scid Il2rg−/− mice
Source: Protein Cell. 2018 Jun 13;10(1):31–42. doi: 10.1007/s13238-018-0558-z (PMC6321811; doi:10.1007/s13238-018-0558-z)
Supplement: Supplementary file 1 — Supplementary material 1 (PDF 60 kb) [file 13238_2018_558_MOESM1_ESM.pdf]

Supplementary Fig. 1:

(A) Genotyping analysis of the NOD-*scid Il2rg*$^{-/-}$ EPS or cEPS cells. ICR: ICR strain EPS cell lines.

(B) Karyotyping analysis of ES cell lines under 2i/LIF condition (passage 22 and 23). Red boxes indicate abnormal chromosome. Each cell line counts 30 cells.

(C) qRT-PCR analysis of pluripotent gene expression in NOD-*scid Il2rg*$^{-/-}$ EPS and cEPS cells. Error bars indicate SEM (n = 2).

(D) Schematic of *Tdtomato* reporter knock-in to label NOD-*scid Il2rg*$^{-/-}$ EPS and cEPS cells.

(E) Representative images of TD^{+} EPS and cEPS colonies after nucleofection with pX330 plasmid and targeting vector. Scale bar, 50 μm.

(F) The bar chart showing the percentage of chimeras with NOD-*scid Il2rg*$^{-/-}$ EPS or cEPS cells contributing to the embryo, yolk sac and placenta in vivo at E13.5. n indicates numbers of E13.5 fetuses.

(G) Images of primary colonies of ICR and 129×OG strains at the end of the chemical induction (day 40) and the cEPS colonies for P9 and P7 passages respectively.

Supplementary Fig. 2:

(A) Schematic of human *IL-6* expression cassette targeting mouse *IL-6* in NOD-*scid Il2rg*$^{-/-}$ EPS cells.

(B) Genomic PCR of human *IL-6* inserted NOD-*scid Il2rg*$^{-/-}$ EPS cells to identify correct targeted colonies. HDR5 and HDR3: homologous arm fragments; Human *IL-6*: human *IL-6* fragments.

(C) Phase-contrast images of human *IL-6* inserted NOD-*scid Il2rg*$^{-/-}$ EPS cells for 15 passages in LCDM medium. Scale bars, 100 μm.

(D) Postnatal chimeras generated by injection of human *IL-6* inserted NOD-*scid Il2rg*$^{-/-}$ EPS cells into C57BL/6 8-cell embryo.

(E) ELISA analysis of human IL-6 level in chimera after LPS stimulation.
