# Supplementary material for: Correction to: Efficient derivation of extended pluripotent stem cells from NOD-scid Il2rg−/− mice
Source: Protein Cell. 2018 Aug 10;10(2):154–5. doi: 10.1007/s13238-018-0571-2 (PMC6340893; doi:10.1007/s13238-018-0571-2)
Supplement: Supplementary file 1 — Electronic supplementary material 1 (PDF 170 kb) [file 13238_2018_571_MOESM1_ESM.pdf]

Supplementary Fig. 1:

(A) Genotyping analysis of the NOD-*scid Il2rg*$^{-/-}$ EPS or cEPS cells. ICR: ICR strain EPS cell lines.

(B) Karyotyping analysis of ES cell lines under 2i/LIF condition (passage 22 and 23). Red boxes indicate abnormal chromosome. Each cell line counts 30 cells.

(C) qRT-PCR analysis of pluripotent gene expression in NOD-*scid Il2rg*$^{-/-}$ EPS and cEPS cells. Error bars indicate SEM (n = 2).

(D) Schematic of *Tdtomato* reporter knock-in to label NOD-*scid Il2rg*$^{-/-}$ EPS and cEPS cells.

(E) Representative images of TD^{+} EPS and cEPS colonies after nucleofection with pX330 plasmid and targeting vector. Scale bar, 50 μm.

(F) The bar chart showing the percentage of chimeras with NOD-*scid Il2rg*$^{-/-}$ EPS or cEPS cells contributing to the embryo, yolk sac and placenta in vivo at E13.5. n indicates numbers of E13.5 fetuses.

(G) Images of primary colonies of ICR and 129×OG strains at the end of the chemical induction (day 40) and the cEPS colonies for P9 and P7 passages respectively.

Supplementary Fig. 2:

(A) Schematic of human *IL-6* expression cassette targeting mouse *IL-6* in NOD-*scid Il2rg*$^{-/-}$ EPS cells.

(B) Genomic PCR of human *IL-6* inserted NOD-*scid Il2rg*$^{-/-}$ EPS cells to identify correct targeted colonies. HDR5 and HDR3: homologous arm fragments; Human *IL-6*: human *IL-6* fragments.

(C) Phase-contrast images of human *IL-6* inserted NOD-*scid Il2rg*$^{-/-}$ EPS cells for 15 passages in LCDM medium. Scale bars, 100 μm.

(D) Postnatal chimeras generated by injection of human *IL-6* inserted NOD-*scid Il2rg*$^{-/-}$ EPS cells into C57BL/6 8-cell embryo.

(E) ELISA analysis of human IL-6 level in chimera after LPS stimulation.

Figure S1

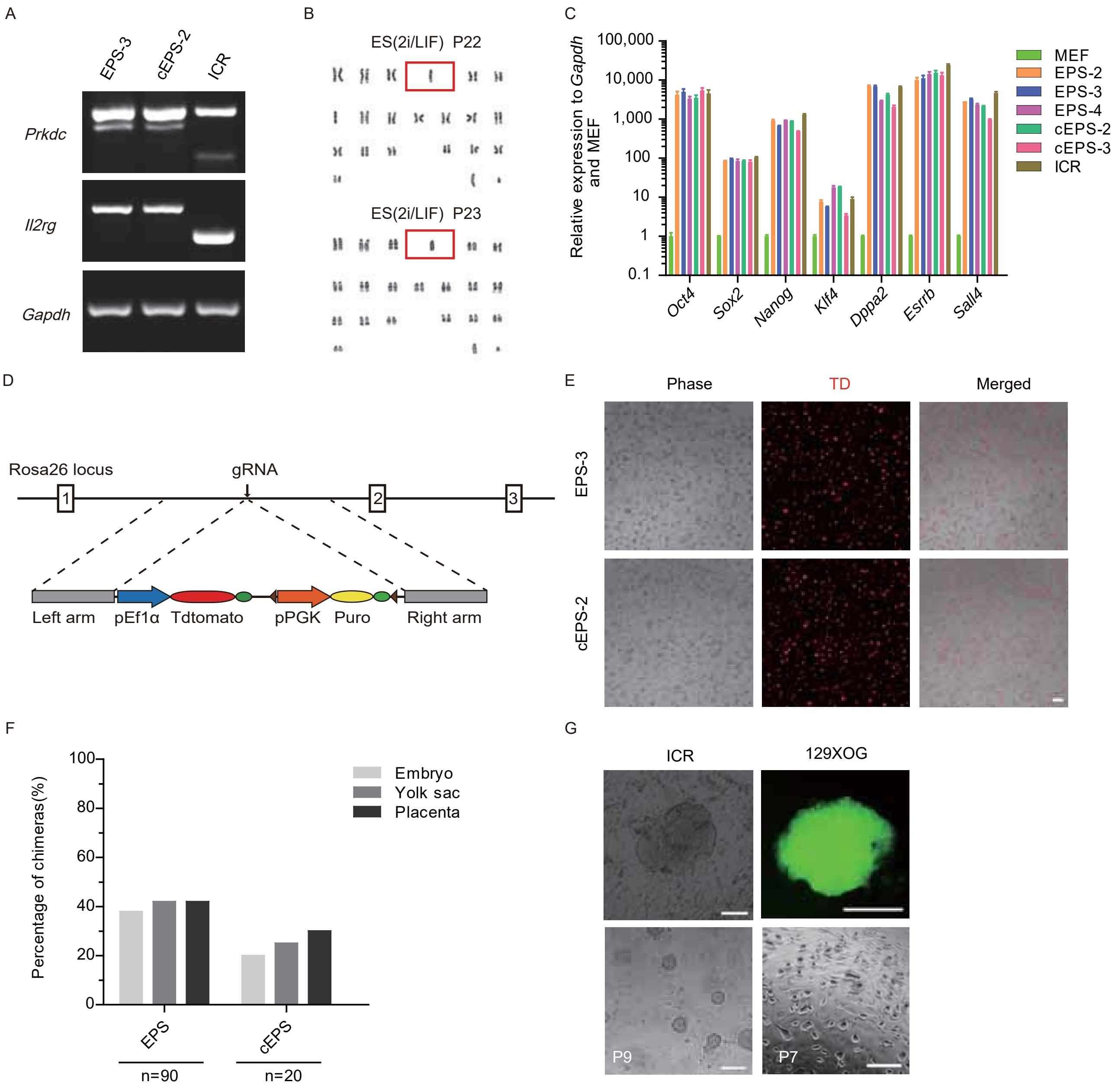

A
EPS-3
cEPS-2
ICR
Prkdc
Il2rg
Gapdh
B
ES(2i/LIF) P22
ES(2i/LIF) P23
C
Relative expression to Gapdh and MEF
100,000
10,000
1,000
100
10
1
0.1
Oct4
Sox2
Nanog
Klf4
Dppa2
Esrrb
Sall4
MEF
EPS-2
EPS-3
EPS-4
cEPS-2
cEPS-3
ICR
D
Rosa26 locus
gRNA
1
2
3
Left arm
pEf1α
Tdtomato
pPGK
Puro
Right arm
E
Phase
TD
Merged
EPS-3
cEPS-2
F
Percentage of chimeras(%)
100
80
60
40
20
0
Embryo
Yolk sac
Placenta
EPS
cEPS
n=90
n=20
G
ICR
129XOG
P9
P7

Figure S2

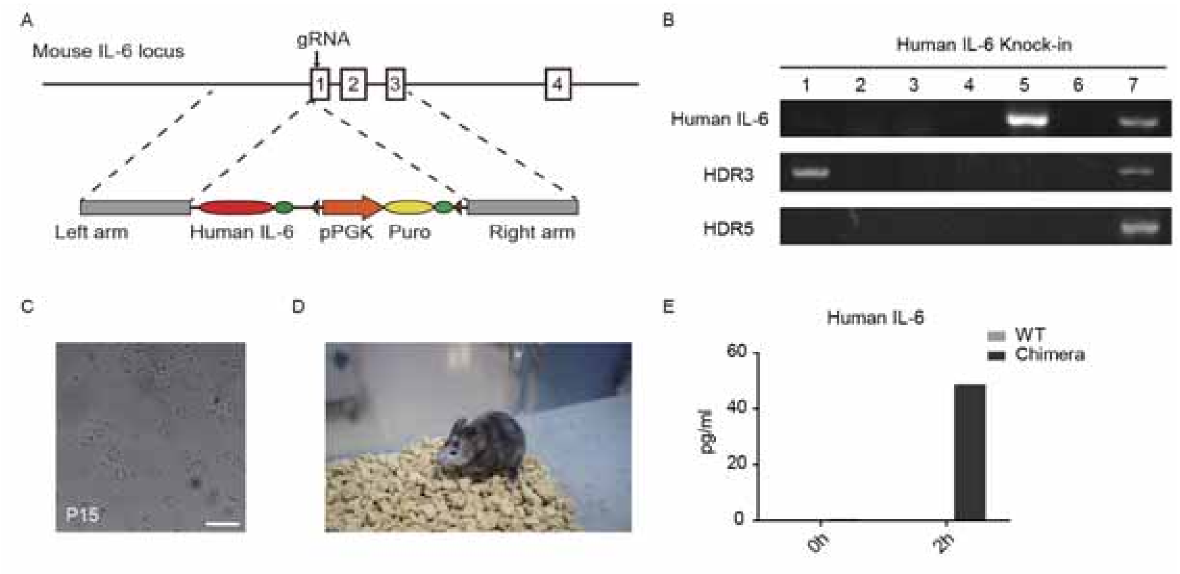
A
Mouse IL-6 locus
gRNA
1
2
3
4
Left arm
Human IL-6
pPGK
Puro
Right arm
B
Human IL-6 Knock-in
1 2 3 4 5 6 7
Human IL-6
HDR3
HDR5
C
P15
D
E
Human IL-6
WT
Chimera
pg/ml
60
40
20
0
0h
2h

Table S1

Primers used in this study

Primers	Sequences
Primers for genotyping genes	
PRKDC-F	GAGAAAAGGAGGATCATGGATTCAAGAAATAAATGTAACG
PRKDC-WR	TGGCCCCTGCTAACTTTCTCTTAGCA
PRKDC-MF	TGGTATCCACAACATAAAATACGCTAA
PRKDC-R	CCTAAGAGTCACTTTCTCCATTTACACAGTGAAGTGCC
IL2RG-Co	GTGGGTAGCCAGCTCTTCAG
IL2RG-WT	CCTGGAGCTGGACAACAAAT
IL2RG-Mu	GCCAGAGGCCACTTGTGTAG
Gapdh-F	CCCACTAACATCAAATGGGG
Gapdh-R	CCTTCCACAATGCCAAAGTT
Primers for XEN marker genes	
Sox17-F	GTCAACGCCTTCCAAGACTTG
Sox17-R	GTAAAGGTGAAAGGCGAGGTG
Gata4-F	GAGCTGGCCTGCGATGTCTGAGTG
Gata4-R	AAACGGAAGCCCAAGAACCTGAAT
Sall4-F	TGGCAGACGAGAAGTTCTTTC
Sall4-R	TCCAACATTTATCCGAGCACAG
Sox7-F	GATGAGAGGAAACGTCTGG
Sox7-R	CTTCCATGACTTTCCCAGC
Gapdh-F	CCCACTAACATCAAATGGGG
Gapdh-R	CCTTCCACAATGCCAAAGTT
Primers for pluripotent marker genes	
Sox2-F	CGGGAAGCGTGTACTTATCCT
Sox2-R	GCGGAGTGGAAACTTTTGTCC
Klf4-F	TTGCGGTAGTGCCTGGTCAGTT
Klf4-R	CTATGCAGGCTGTGGCAAAACC
Oct4-F	CAGGGCTTTCATGTCCTGG
Oct4-R	AGTTGGCGTGGAGACTTTGC
Nanog-F	AGTTATGGAGCGGAGCAGCAT
Nanog-R	AGGCCTGGACCGCTCAGT
Dppa2-F	GCGTAGCGTAGTCTGTGTTTG
Dppa2-R	TCAACGAGAACCAATCTGAGGA
Sall4-F	TGGCAGACGAGAAGTTCTTTC
Sall4-R	TCCAACATTTATCCGAGCACAG
Esrrb-F	GTGGCTGAGGGCATCAATG
Esrrb-R	AACCGAATGTCGTCCGAAGAC
Gapdh-F	CCCACTAACATCAAATGGGG
Gapdh-R	CCTTCCACAATGCCAAAGTT
Primers for placenta specific genes	
Furin-F	TGCCAGACCACATGACTAC
Furin-R	CAAGGACTTGGGGGATGAA
Pl1-F	TTGGCCGCAGATGTGTATAG
Pl1-R	TCGTGGACTTCCTCTCGATT
Hand1-F	ATGAACCTCGTGGGCAGGTA
Hand1-R	TCACTGGTTTAGCTCCAGCG
Plf-F	TCCTGGATACTGCTCCTACTACT
Plf-R	GACCATTCCTCATTGCACACA
Tpbpa-F	CACAGTAGCGAAAATGACCAGG
Tpbpa-R	TCCTCCTCTTCAAACATTGGGT
Ctsq-F	CATTGCCAGTTGACAACACAAG
Ctsq-R	ATAGCCTTCATTTCGCCAATCA
Pl2-F	CCAACGTGTGATTGTGGTGTC
Pl2-R	CAGGCCATAGGTCCAAGCTG
Gapdh-F	CCCACTAACATCAAATGGGG
Gapdh-R	CCTTCCACAATGCCAAAGTT
Primers for genomic targeting	
G-Test-F	GCCTTTATGCCTTTAATCCCAGCACTCT
G-Test-R	ATTTTACCAACTATCACCCAAGCTCCCT
Ef1a-Td-F	GCCACTATGGGGTACGGCTCCGGTGCCCGTCAG
Ef1a-Td-R	CCATGGTGGCCTCGATCACGACACCTGAAATGGA
Puro-F	GCAATTGTTGTTGTTGCGGCCGCGACTCTAGAT
Puro-R	ATCAGCGAGCTCTAGGCATCCCGGGGATCTGAT
HDR5-F	TGGATGTATGCTCCCGACTT
HDR5-R	TTCTGCCAGTGCCTCTTTGC
HDR3-F	CTCTTTACTGAAGGCTCTTTACTATTGCT
HDR3-R	TCCACTTCTGACCCTCACTCCTT
Human IL-6-F	CACAGACAGCCACTCACCTC
Human IL-6-R	AGGCTGGCATTTGTGGTTGG

Table S2

Antibodies used in this study

Antibodies	Company	Catalog Number
Anti-Oct-3/4 (C-10)	Santa Cruz Biotechnology	sc-5279
Anti-Sox-2 (Y-17)	Santa Cruz Biotechnology	sc-17320
Anti-Cytokeratin 8 (M20)	Santa Cruz Biotechnology	sc-52324
Anti-β3 Tubulin (AA10)	Santa Cruz Biotechnology	sc-80016
Anti-Proliferin(E-10)	Santa Cruz Biotechnology	sc-271891
Anti-α-Actinin	Sigma-Aldrich	A7811
Anti-Trophoblast specific protein alpha	Abcam	ab104401
Anti-FOXA2	Abcam	ab60721
Anti-TBR2/Eomes	Abcam	ab23345
Anti-Sall4 antibody	Abcam	ab29112
Anti-Stage-Specific Embryonic Antigen-1	Millipore	MAB4301
Anti-Nanog	R&D	AF2729
Anti-Sox17	R&D	AF1924
Anti-Gata6	R&D	AF1700
Alexa Fluor 488-AffiniPure Donkey Anti-Mouse IgG (H+L)	Jackson ImmunoResearch	715-545-150
Alexa Fluor 488-AffiniPure Donkey Anti-Rabbit IgG (H+L)	Jackson ImmunoResearch	711-545-152
Alexa Fluor 488-AffiniPure Donkey Anti-Goat IgG (H+L)	Jackson ImmunoResearch	705-545-147
Alexa Fluor 647 Donkey Anti-Rabbit IgG (H+L)	Jackson ImmunoResearch	711-605-152
Cy3-AffiniPure Donkey Anti-Mouse IgG (H+L)	Jackson ImmunoResearch	715-165-150
Cy3-AffiniPure Donkey Anti-Rabbit IgG (H+L)	Jackson ImmunoResearch	711-165-152

Table S3

Small molecules used in chemical reprogramming

Small molecules	Company	Catalog Number
Valproic acid sodium salt(VPA)	Sigma-Aldrich	P4543
CHIR99021	WUXI APPTEC	N/A
616452	WUXI APPTEC	N/A
Tranylcypromine	Enzo	BML-EI217-0005
Forskolin	Enzo	BML-CN100-0100
AM580	Tocris	0760
EPZ004777	Selleckchem	S7353
SGC0946	Selleckchem	S7079
3-deazaneplanocin(DZNep)	WUXI APPTEC	N/A
5-Aza-2'-deoxycytidine(5-aza-dC)	Enzo	ALX-480-096-M005
PD0325901	Selleckchem	S1036
