# Supplementary material for: Correction to: Efficient derivation of extended pluripotent stem cells from NOD-scid Il2rg−/− mice
Source: Protein Cell. 2018 Aug 10;10(2):154–5. doi: 10.1007/s13238-018-0571-2 (PMC6340893; doi:10.1007/s13238-018-0571-2)
Supplement: Supplementary file 2 — Electronic supplementary material 2 (PPTX 62 kb) [file 13238_2018_571_MOESM2_ESM.pptx]

## Slide 1
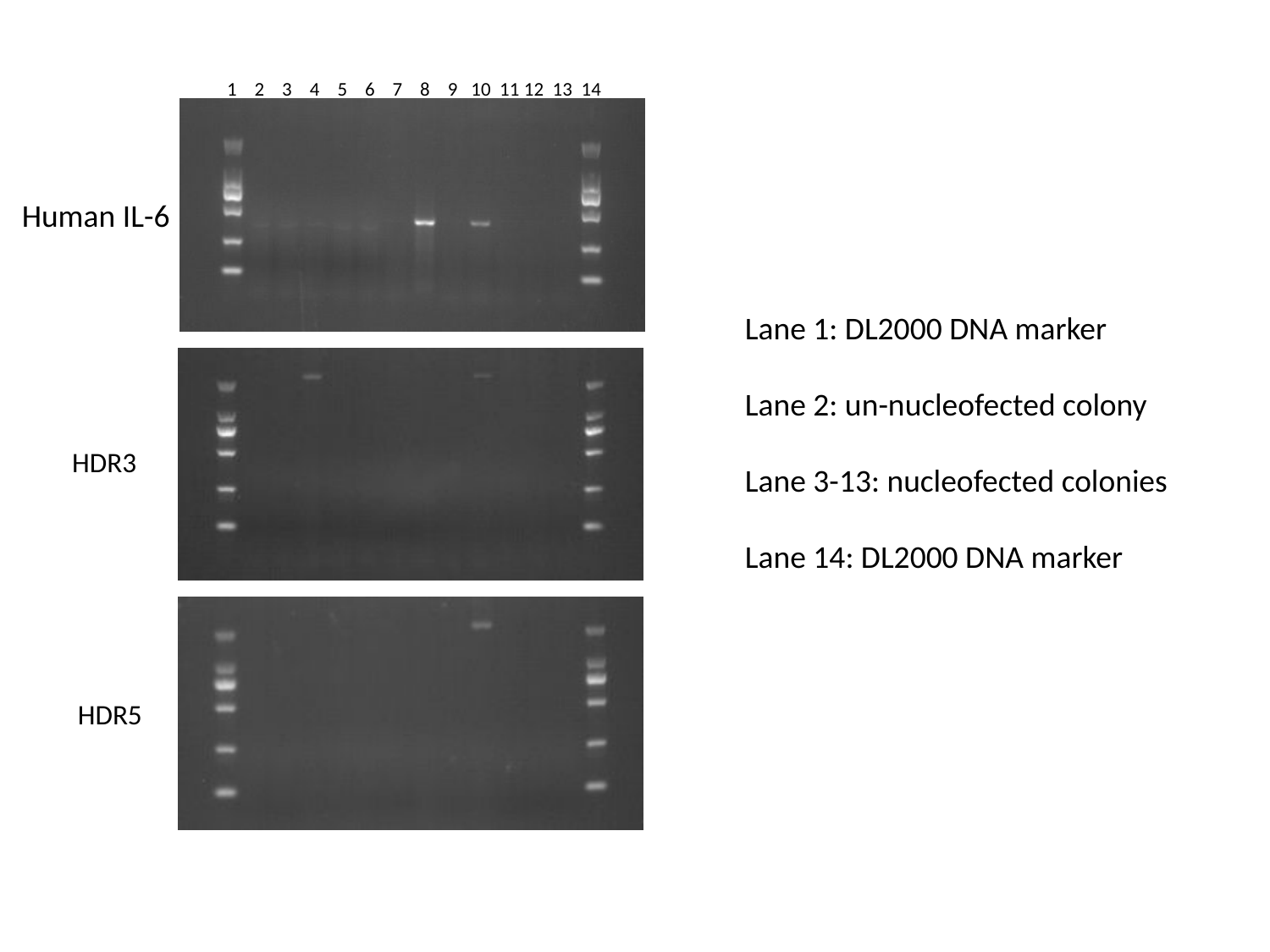

1 2 3 4 5 6 7 8 9 10 11 12 13 14
Human IL-6
Lane 1: DL2000 DNA marker
Lane 2: un-nucleofected colony
Lane 3-13: nucleofected colonies
Lane 14: DL2000 DNA marker
HDR3
HDR5
